# Adult-onset neuronal ceroid lipofuscinosis misdiagnosed as autoimmune encephalitis and normal-pressure hydrocephalus: A 10-year case report and case-based review

**DOI:** 10.1097/MD.0000000000040248

**Published:** 2024-10-25

**Authors:** Huasheng Huang, Yuqi Liao, Yanni Yu, HuiHui Qin, Yi Zhi Wei, Liming Cao

**Affiliations:** a Department of Neurology, Liuzhou People’s Hospital, Liuzhou, Guangxi Zhuang Autonomous Region, China; b School of Medicine, Shenzhen University, Shenzhen, Guangdong Province, China; c Department of Neurology, The First Affiliated Hospital of Shenzhen University, Shenzhen, China; d Hunan Provincial Key Laboratory of the Research and Development of Novel Pharmaceutical Preparations, Changsha Medical University, Changsha, Hunan Province, China.

**Keywords:** adult-onset neuronal ceroid lipofuscinosis, electron microscopy, genetic test, neuroimaging characteristics, refractory epilepsy

## Abstract

**Rationale:**

Neuronal ceroid lipofuscinoses (NCLs) are rare, fatal, inherited neurodegenerative disorders characterized by myoclonic epilepsy, cognitive decline, brain atrophy, and retinopathy. The pathogenesis and clinical manifestations of NCL are not well understood and frequently result in misdiagnosis and overtreatment. The aim of this case report and review is to improve our understanding of the clinical features and management of NCL.

**Patient concerns:**

A 36-year-old woman initially presented with refractory epilepsy.

**Diagnoses:**

Initially diagnosed with autoimmune encephalitis, the patient was later diagnosed with normal-pressure hydrocephalus. A definitive diagnosis of adult-onset neuronal ceroid lipofuscinosis (ANCL) was established after 10 years of observation, utilizing biopsy and genetic testing.

**Interventions:**

High-dose intravenous immunoglobulin and methylprednisolone were administered, along with the insertion of a ventriculoperitoneal shunt.

**Outcomes:**

Despite various treatments, the patient’s condition did not improve.

**Lessons:**

ANCL typically presents with the clinical triad of refractory seizures, progressive cognitive decline, and movement disorders. Neuroimaging often reveals progressive brain atrophy on magnetic resonance imaging, while electroencephalograms frequently show epileptiform discharges. The prognosis is generally poor. Improved understanding of ANCL from both clinical and radiological perspectives, coupled with early consideration of differential diagnoses, could minimize unnecessary interventions and optimize patient care.

## 1. Introduction

Neuronal ceroid lipofuscinosis (NCL) is a group of rare, fatal, inherited neurodegenerative lysosomal storage disorders.^[1]^ NCL exhibits a high degree of genetic and clinical heterogeneity, with characteristic pathological changes, including intraneuronal wax-like or lipofuscin-like deposits of autoimmune fluorescent substances.^[2,3]^ The prevalence of NCL is approximately 1 in 12,500 in Europe and North America,^[4]^ with an estimated global incidence of 1 in 100,000.^[5]^ Common symptoms of NCL include progressive vision loss, mental and motor deterioration, epileptic seizures, and dementia.^[1,2]^ Based on the age of onset and clinical features, NCL is classified as congenital, infantile, late infantile, juvenile, or adult-onset NCL (ANCL).^[1]^ ANCL, also known as Kufs disease, is a rare form of NCL that has an onset in adulthood. Two clinical Kufs phenotypes have been described: 1 featuring generalized tonic-clonic seizures and the other characterized by dementia.^[6]^ NCL generally begins in childhood, with an earlier onset associated with a more severe clinical course and prognosis. Early diagnosis is crucial to initiate therapy in asymptomatic patients. However, the early diagnosis of NCL is challenging.

Herein, we describe the case of a 36-year-old woman who initially presented with refractory epilepsy mimicking autoimmune encephalitis and normal-pressure hydrocephalus. After 10 years and 5 hospitalizations, a comprehensive assessment of the clinical symptoms, electron microscopy findings, and genetic tests finally confirmed the diagnosis of ANCL. This case report and review aim to improve our understanding of the clinical features and management of the disease.

## 2. Case presentation

A 36-year-old Han Chinese woman was hospitalized in June 2011 after loss of consciousness and generalized convulsions. She had no history of genetic diseases, and her medical history was unremarkable. Neurological examination revealed no abnormalities, and brain magnetic resonance imaging (MRI) findings were normal. She was initially diagnosed with generalized tonic-clonic epilepsy and experienced seizures every 4 to 5 months for the next 6 years while taking sodium valproate.

**Figure 1.**
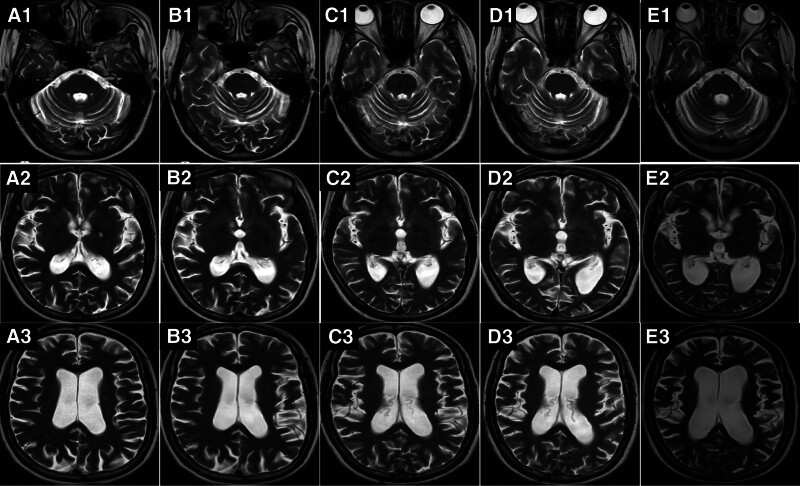
T2-weighted imaging follow-up results of the patient from 2017 to 2023. magnetic resonance imaging (MRI) data for 2017, 2018, 2019, 2020, and 2021 are coded as A1–A3, B1–B3, C1–C3, D1–D3, and E1–E3, respectively.

Six years after the first episode, the patient was readmitted due to a seizure. Physical examination revealed memory impairment, reduced facial expression, mildly increased limb muscle tone, occasional mild tremors, and an unstable gait. The Hasegawa Dementia Scale indicated dementia. Brain MRI and magnetic resonance spectroscopy showed brain atrophy and reduced hippocampal neurons (Fig. 1A1–A3). The electroencephalogram displayed widespread slow waves and epileptic wave discharges. The patient was diagnosed with epilepsy, Parkinson syndrome, and dementia. She was administered sodium valproate 1 g/d, lamotrigine 0.5 mg/d, benzhexol 4 mg/g, and levodopa 0.375 g/d, and based on the patient’s response to the medication, the dosage of the antiepileptic drugs was gradually increased. However, no improvement was observed in cognitive and motor functions. One year after discharge, follow-up brain MRI showed a slight worsening of brain atrophy (Fig. 1B1–B3).

Eight years after symptom onset, the patient was admitted for the third time because of repeated tremors in the upper limbs and trunk for over 2 years. Physical examination revealed impaired memory, slurred speech, mildly increased limb muscle tone, and instability in the finger-to-nose test. The Mini-Mental State Examination score was 8 points, indicating severe cognitive impairment; brain MRI demonstrated brain atrophy (Fig. 1C1–C3); cerebrospinal fluid (CSF) analysis was normal; and antibodies for autoimmune encephalitis, anti-myelin oligodendrocyte glycoprotein, and anti-aquaporin-4 in the blood and CSF were negative. The patient was diagnosed with autoimmune encephalitis, Parkinson syndrome, and dementia. High-dose intravenous immunoglobulin and methylprednisolone were administered once each, followed by oral prednisone and mycophenolate mofetil for approximately 1 year. The patient was prescribed lamotrigine, sodium valproate, clonazepam, benzhexol, idebenone, and donepezil. However, no significant posttreatment improvement was observed.

Nine years after the initial onset, the patient was admitted for the fourth time due to repeated limb tremors for 3 years. Physical examination revealed impaired memory, unclear speech, generalized tremors (muscle spasm attacks), mildly increased limb muscle tone, a festinating gait, and a positive finger-to-nose test. Blood analysis showed an antinuclear antibody titer of 1:100 (reference range: negative), anti-Sjögren’s syndrome A antibody positivity (reference range: negative), and elevated lactate (4.0 mmol/L, reference range: 0.5–2.2 mmol/L), thyroid peroxidase antibody (219.9 IU/L, reference range: 0–60 IU/L), thyroid stimulating hormone (9.45 MIU/L, reference range: 0.35–4.94 MIU/L), and anti-thyroglobulin antibody (513.1 IU/L, reference range: 0–40 IU/L) levels. Brain MRI exhibited worsening brain atrophy (Fig. 1D1–D3). Sublabial gland biopsy did not confirm Sjögren’s syndrome. Thus, the patient was diagnosed with Hashimoto thyroiditis and hypothyroidism, for which levothyroxine was prescribed. Despite treatment with sodium valproate, lamotrigine, and levodopa, her symptoms did not improve.

**Figure 2.**
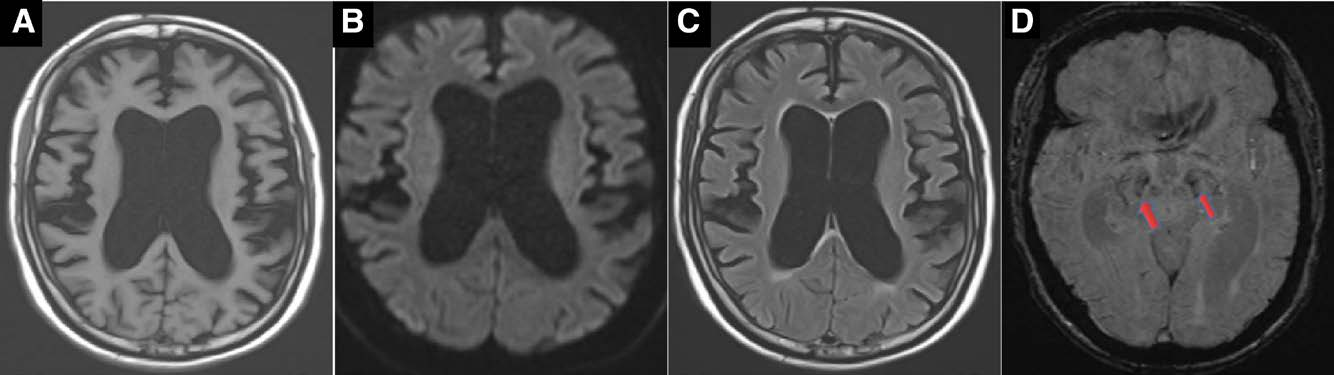
Brain MRI. Substantial ventricular enlargement and cerebral atrophy on T1-weighted images (A), diffusion-weighted images (B), and fluid-attenuated inversion recovery sequences (C), with susceptibility-weighted images displaying indistinct bilateral substantia nigra (D, arrows).

**Figure 3.**
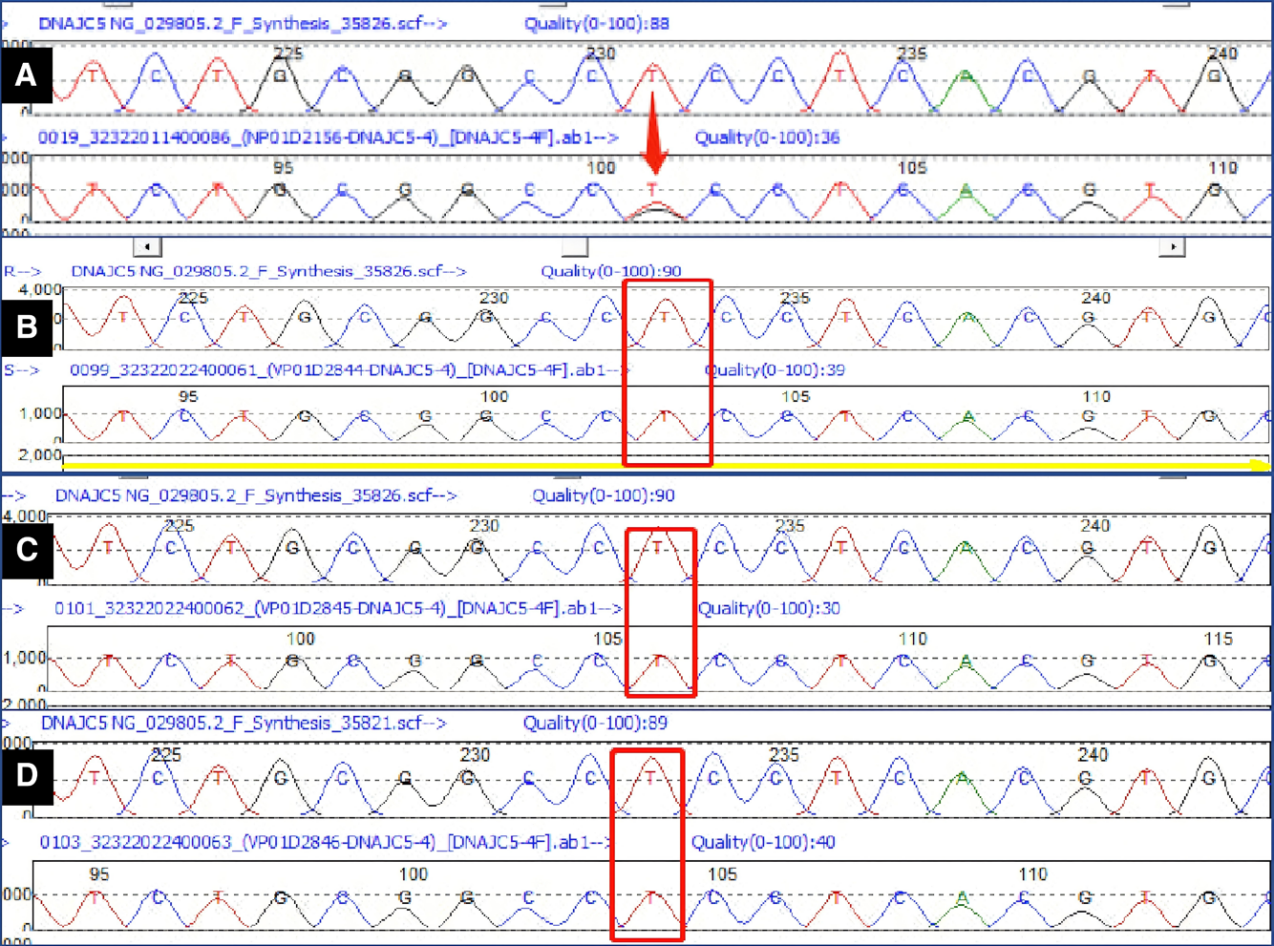
Genetic sequencing reveals a heterozygous mutation in *DNAJC5*. (A) The heterozygous missense mutation (c.344T > G, arrow) in the proband. (B–D) correspond to the control sequences of the proband’s unaffected father, mother, and son, respectively.

**Figure 4.**
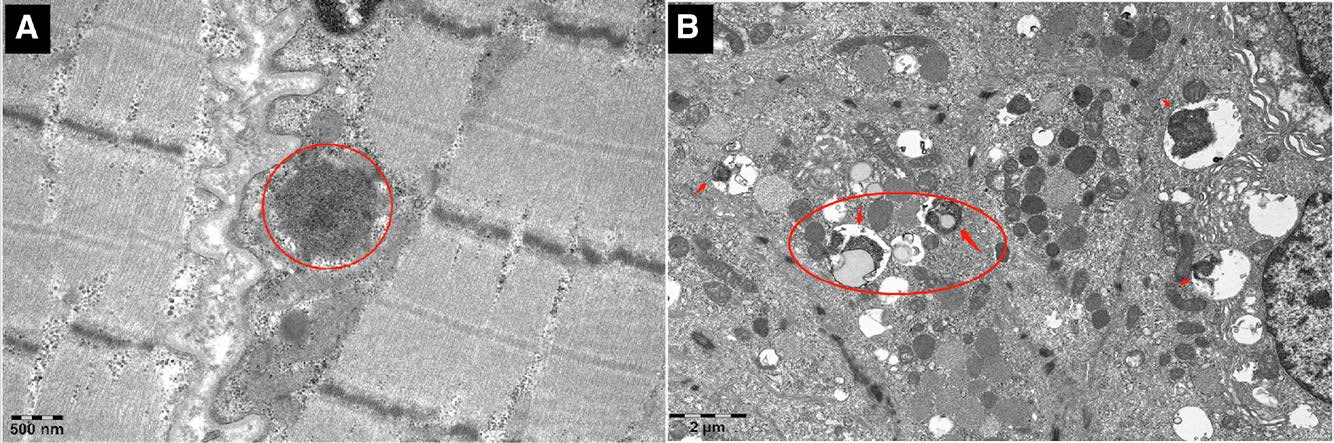
Electron microscopy pathological findings from skin biopsy. (A) Muscle biopsy shows a small number of granular osmiophilic deposits beneath the sarcolemma. (B) Skin biopsy reveals abundant granular osmiophilic deposits and lipofuscin within the cytoplasm of glandular epithelial cells.

A decade after the initial episode, the patient was hospitalized for the fifth time owing to repeated seizures and motor deficits. Seizure episodes markedly diminished; however, the patient was self-care deficient. Examination revealed cognitive decline akin to dementia, facial hypomimia, dysarthria, and hypertonia in the extremities. Rechecking for antibodies for autoimmune encephalitis, paraneoplastic syndrome, and central nervous system demyelinating diseases produced negative results. Repeated CSF analysis results were normal. Enhanced MRI of the head indicated substantial ventricular enlargement and brain atrophy (Figs. 1E1–E3 and 2A–C), with susceptibility-weighted (SW) imaging showing blurred bilateral substantia nigra (Fig. 2D). Routine fundus examination revealed no significant abnormalities. The possibility of normal-pressure hydrocephalus was not ruled out. There was an obvious improvement in gait after the CSF drainage test, leading to the implementation of a ventriculoperitoneal shunt; nevertheless, the patient’s condition did not improve further. The potential of neurogenetic disorders persisted. Whole-exome sequencing of DNA from peripheral blood samples revealed a novel missense mutation (c.344T > G [p.Leu115Arg]) in *DNAJC5* (Fig. 3), which was absent in the patient’s parental blood samples. Electron microscopic pathological diagnosis of the skin and skeletal muscle (Ultrastructural Pathology Center, Renmin Hospital of Wuhan University) revealed NCL (Fig. 4A and B). A comprehensive assessment of the clinical symptoms, electron microscopy findings, and genetic tests confirmed the diagnosis of ANCL.

### 2.1. Standard protocol approvals, registrations, and patient consent

This study was approved by the ethics review board of Liuzhou People’s Hospital (No. 2023 KY-E-02) and followed the ethical standards of the 1964 Declaration of Helsinki and its subsequent amendments. Written informed consent for publication was obtained from the participant’s parent.

## 3. Literature review

### 3.1. Search strategy

A literature search was performed using the PubMed database from January 1, 1990, to April 1, 2024. The initial search strategy for publications was conducted by 2 authors using the search terms (Lysosomal Storage Disorders OR Kufs Disease OR NCL OR Parry Disease) and (Adult-onset), OR (Adult-onset NCL) OR (Adult-onset Kufs Disease) OR (ANCL) OR (Adult type of neuronal ceroid lipofuscinosis) OR (Adult neuronal ceroid lipofuscinosis). No restrictions, limits, or filters were applied.

### 3.2. Inclusion/exclusion criteria

Case reports, case series, original research, and case reports with literature reviews were included in the study. Only patients diagnosed with ANCL through pathological or genetic testing were considered. Two reviewers independently analyzed the full texts and reviewed citation lists in the included articles for additional references; any discrepancies were resolved through negotiation or consultation with the corresponding author. Titles and abstracts were screened, and full-text publications were assessed for further inclusion or exclusion. Duplicate references and redundant publications were removed. Cases with substantial deficiencies in medical records were excluded from the study.

### 3.3. Results

We ultimately identified 15 case reports of ANCL confirmed through pathology.^[6–13]^ From each article, we extracted data including the first author, patient age, sex, MRI, and electroencephalogram results, and follow-up information. The clinical and imaging characteristics of ANCL cases documented in the literature are summarized in the Table 1.

**Table 1:** Summary of the reported clinical features of diagnosed adult-onset neuronal ceroid lipofuscinosis confirmed by pathology.

	Age (yr), sex	Main symptoms	Brain CT or MRI	EEG results	Treatment	Follow-up results
Josephson et al^[6]^	32, F	Forgetfulness, progressive weakness of the left leg, and speech difficulties	MRI was unremarkable	Frequent spike activity and 1 electrical seizure	Not reported	Progressive cognitive and physical deterioration eventually led to an inability to live independently
Josephson et al^[6]^	40, F	Seizures, visual aura, choreiform movements, and memory difficulties	Repeated head CT showed generalized atrophy	Paroxysmal activity with a left temporal focus	Not reported	The patient was bedridden, mixed aphasia, and needed complete care
Josephson et al^[6]^	35, F	GTCS, followed by forgetfulness	Head CT showed no abnormalities, while the subsequent MRI revealed brain atrophy	Generalized epileptiform activity	Not reported	Not reported
Canafoglia et al^[7]^	22, M	Visual loss, GTCS, mild ataxia, mild depression, and palinopsia	MRI revealed severe cerebellar atrophy	Moderate paroxysmal activity (small spikes and waves)	Levetiracetam and valproate for seizure management	Seven years after the onset, the patient experienced severe visual impairment but remained able to work
Canafoglia et al^[7]^	23, F	GTCS, follow by minimal ataxia, visual impairment, palinopsia, and mild depression	MRI revealed severe cerebellar atrophy	Moderate paroxysmal activity (small spikes and waves)	Levetiracetam effectively managed epilepsy	Atrophic retinal changes became obvious 2 years later
Smith et al^[8]^	20, F	Tremor, followed by ataxia and dysarthria, ultimately leading to dementia	MRI showed cortical and cerebellar atrophy	Not reported	Not reported	The patient died at the age of 42 years
Smith et al^[8]^	24, F	Focal seizures, followed by dementia, mood disturbances, tremor, and ataxia	MRI showed diffuse atrophy	Not reported	Not reported	Institutional care was necessary, and the patient required a wheelchair
Nosková et al^[9]^	34, M	GTCS, followed by progressive confusion and dementia	MRI showed diffuse cerebral and cerebellar atrophy	Generalized periodic epileptiform discharges superimposed on a background of diffuse low-amplitude spikes	Not reported	The patient was wheelchair bound and required nursing-home care
Di Fabio et al^[10]^	43, F	Tonic-clonic seizures, rapid cognitive decline, and postural tremor	MRI showed atrophy in the parieto-occipital lobes and cerebellum and hyperintensities in the periventricular areas	Not reported	Sodium valproate and carbamazepine	Not reported
Di Fabio et al^[10]^	45, F	Tonic-clonic seizures, cognitive impairment, ideomotor apraxia, cerebellar dysarthria, and abnormal behavior	MRI showed atrophy in the cortico-subcortical regions, cerebellum, and corpus callosum and hyperintensities in the periventricular white matter	Not reported	Sodium valproate, phenobarbital, and zonesamide	Not reported
Ivan et al^[11]^	27, F	Dysarthria, rigidity in both lower extremities, memory loss, followed by ataxia, extreme aggressiveness, and dementia	MRI showed generalized mild atrophy, along with a slight increase in the T2 signal in the periventricular area	Sluggish posterior rhythm, slow overall activity, and widely dispersed extremely low voltage activity	Not reported	The patient died at the age of 46 years
Berkovic et al^[12]^	30, F	GTCS, myoclonus, followed by dementia	CT showed minimal generalized atrophy	Generalized spike and slow wave complexes and polyspike and wave complexes	Phenytoin, carbamazepine, clonazepam, barbiturates, valproic acid, and carbidopa	The treatment did not achieve lasting improvements
Berkovic et al^[12]^	31, F	Myoclonus, GTCS, and progressive cognitive impairment	Not reported	Epileptiform discharges were uncommon; photic stimulation elicited a notable photoparoxysmal response	Valproate and clonazepam appeared to achieve the best treatment effects	Not reported
Özkara et al^[13]^	18, F	Initial GTCS, followed by refractory epilepsy seizures	The initial MRI was normal Follow-up MRI showed significant global cortical atrophy	Bitemporal sharp waves	Carbamazepine, followed by valproate, and finally piracetam	After piracetam administration, there was a notable improvement in gait ataxia
Özkara et al^[13]^	26, M	GTCS, myoclonia, extremity ataxia, dysarthria	The initial MRI was normal Follow-up MRI showed mild atrophy	Not reported	Valproate, piracetam, and topamoxate	Myoclonia was repeatedly observed

Abbreviations: CT = computed tomography, EEG = electroencephalography, F = female, GTCS = generalized tonic-clonic seizures, M = male, MRI = magnetic resonance imaging.

Patients typically develop the disease between the ages of 18 and 45, with a higher prevalence among young and middle-aged adults, and it mostly affects females. Key clinical manifestations include epileptic seizures (especially generalized tonic-clonic seizures), cognitive impairments (including memory decline and dementia), motor disturbances (such as muscle spasms, ataxia, tremors, and limb weakness), and visual impairments.

Most patients exhibit varying degrees of brain atrophy on computed tomography or MRI scans, including cortical and cerebellar atrophy. Some cases also show high signal changes in the periventricular white matter. Electroencephalograms typically show epileptiform discharges like sharp waves, spike waves, and slow waves, signifying abnormal brain activity.

Treatment primarily includes antiepileptic drugs, such as sodium valproate, carbamazepine, and levetiracetam. However, their effectiveness is limited, making it difficult to stop the progression of the disease. For most patients, the outlook is grim, as progressive decline results in substantial cognitive and motor deficits, ultimately leading to the loss of independent living ability and the need for constant care. In some instances, symptoms may partially improve with antiepileptic medication, but functional impairments still endure to some extent.

## 4. Discussion

This report highlights a unique case of ANCL that was identified after a decade-long observation period. The patient presented with intractable epilepsy, Parkinsonism, ataxia, and cognitive deterioration. MRI findings showed severe, progressive brain atrophy with concurrent hydrocephalus. Initially misinterpreted as seronegative autoimmune encephalitis and normal-pressure hydrocephalus, ANCL was eventually considered in the differential diagnosis, with genetic analysis and tissue biopsy being pivotal for the definitive diagnosis. This case was followed continuously for 10 years, highlighting the changes in the patient’s symptoms, signs, and neuroimaging characteristics, which are not available for other similar cases (Table 1). Furthermore, SWI shows a reduction in the substantia nigra, which has not been reported previously.

### 4.1. Pathogenesis of neuronal ceroid lipofuscinosis

The pathogenesis of ANCL involves mutations in several genes, including *CLN3*, *CLN5*, *CLN6*, and *CLN8* that disrupt lysosomal function and affect lipid metabolism and the clearance of cellular waste. Abnormal accumulation of lipofuscin occurs gradually in neurons, ultimately causing neuronal death. A frameshift mutation in the *ATP13A2* gene has been identified as a cause of ANCL in certain dog breeds.^[14]^ Currently, 14 NCL genes have been identified; however, it is unknown whether they act in common disease pathways.^[15]^ A single gene mutation can present with different clinical subtypes, whereas the same clinical phenotype can be caused by different gene mutations. The genetic types of ANCL include *CLN1*, *CLN3*, *CLN4* (*DNAJC5*), *CLN6*, *CLN10*, *CLN11*, and *CLN13*.^[7]^ To date, *DNAJC5*, *CLN6*, and *CLN13* have been reported as pathogenic genes for ANCL, with *DNAJC5* and *CLN6* linked to type A ANCL and *CLN13* linked to type B ANCL.^[8]^ Mutations in *DNAJC5*, which encodes the presynaptic co-chaperone cysteine string protein alpha (CSPα), have been shown to cause autosomal-dominant NCL.^[8]^ Functionally, *DNAJC5* has been implicated in chaperoning synaptic proteins and misfolding-associated protein secretion, but how *DNAJC5* dysfunction causes lipofuscinosis and neurodegeneration is unclear.^[16]^ Palmitoylation-induced aggregation of mutant CSPα proteins may underlie the development of ANCL in affected families.^[17]^ Whole-exome sequencing of the patient in the current report revealed a heterozygous variant of *DNAJC5*, and neither parent of the proband harbored the mutation. Mutations in *DNAJC5* have recently been reported in sporadic adult-onset cases and families with dominant inheritance.^[18]^ The mutation observed in our patient is likely a pathogenic variant that has been identified in several ANCL patients.^[19]^ The leucine residues at positions 115 and 116 are hotspots for mutations and result in a homogeneous phenotype of progressive myoclonic epilepsy with onset at approximately 30 years of age.^[19]^

### 4.2. Clinical features of adult-onset neuronal ceroid lipofuscinosis

ANCL, a dominant-inherited neurodegenerative disease, has an age of onset in the third decade of life^[9]^ and usually begins in the fourth decade of life with seizures.^[6]^ ANCL presents with considerable clinical heterogeneity and common features, such as refractory epilepsy, advancing dementia, behavioral abnormalities, and ataxia.^[7,18]^ Symptoms may vary based on the mutation, especially in terms of severity and age of onset. Seizures are typically the first symptom.^[7,10]^ Unlike childhood NCL, patients with ANCL typically have normal vision.^[10,19,20]^ The patient in this case study presented with adult-onset refractory epilepsy, followed by Parkinsonian symptoms, ataxia, cognitive decline, and ultimately, dementia. Dementia is the primary disabling feature of ANCL, with motor abnormalities being frequent.^[6]^ Cognitive impairments are associated with brain atrophy and intractable epilepsy. Electroencephalography revealed an increase in diffuse slow waves, indicating severe brain damage. Brain atrophy and a reduction in the substantia nigra may lead to muscle tone disorders manifesting as Parkinsonian symptoms. The presence of Parkinsonian symptoms in ANCL has not received sufficient attention, but we believe these symptoms are an important clinical feature of ANCL. ANCL is divided into A and B subtypes. Type A mainly presents as intractable myoclonic epilepsy with dementia, ataxia, and late-onset corticospinal and extrapyramidal symptoms. Type B typically manifests with behavioral abnormalities, dementia, motor dysfunction, ataxia, and extrapyramidal and brainstem-related symptoms.^[3,6,21]^ Patients usually die approximately 15 years after onset.^[9,20,21]^ Earlier onset is linked to more severe symptoms and worse prognosis, with severe neurological damage and secondary multi-organ failure being the most common causes of death.

### 4.3. Magnetic resonance imaging characteristics of adult-onset neuronal ceroid lipofuscinosis

Magnetic resonance imaging can help determine the extent of brain damage. MRI findings from individuals with ANCL may reveal brain atrophy, enlargement of the lateral ventricles, and abnormal signals in the cortical and subcortical areas. The patient’s MR images in our case study showed progressive, extensive, symmetrical whole-brain atrophy. MRI for ANCL typically shows brain atrophy; it is not highly specific. However, some cases show MRI results indicating selective severe cerebellar atrophy.^[7]^ The radiological features of NCL include: atrophy of the cerebral hemisphere cortex and white matter, and (or) various degrees of cerebellar atrophy, which can be diffuse or focal^[10,22]^; and visible brain white matter lesions in the periventricular areas, appearing as multiple patchy high signals on T2-weighted images and as an iso- or low signal on T1-weighted images,^[10,11,23]^ which may be related to lipid deposition in neurons and glial cells. In our patient, no obvious abnormally high signals were observed on T2-weighted images, and the “swallow tail” was not evident on SW images, which may be associated with Parkinsonian symptoms.^[24]^ A striking loss of neurons in the substantia nigra has been observed in patients with ANCL.^[20,21]^ As the condition progresses, brain atrophy becomes more noticeable. Follow-up MRI can be used to monitor disease progression and indicate severity.

### 4.4. *Diagnosis and differentiation of* adult-onset neuronal ceroid lipofuscinosis

The diagnosis of ANCL is based on clinical presentation, family history, neuroimaging, pathology, and genetic testing. Clinicians consider the possibility of ANCL based on clinical presentation, genetic history, and neuroimaging, whereas pathology and genetic testing help confirm the diagnosis and identify the subtype. Middle-aged or young adults with intractable epilepsy, progressive cognitive impairment, Parkinsonian symptoms, and ataxia whose MR images show progressive brain atrophy should be considered for the differential diagnosis of ANCL.^[6,12]^ Pathological examination revealing the accumulation of lipofuscin granules in cells is the gold standard for the diagnosis of NCL. Electron microscopy has shown ample intraneuronal granular osmiophilic deposits.^[22]^ Herein, the patient’s skin pathology showed lipofuscin-like granules, suggesting NCL. Genetic testing, a noninvasive method, has become an important means of diagnosing NCL.^[25]^ In our patient, genetic sequencing revealed a heterozygous mutation in *DNAJC5*, supporting the diagnosis of ANCL. Hence, genetic testing, particularly prenatal, is important.

The diagnosis of ANCL remains challenging; expert pathologic analysis and recent molecular genetic advances have revealed misdiagnoses in over 1-third of cases.^[3]^ The patient in the present report was initially misdiagnosed as having autoimmune encephalitis. Due to the poor treatment response and prominent Parkinsonian symptoms with the lack of the “swallow tail” on SW images, a diagnosis of Parkinson syndrome was made. Subsequently, substantial brain ventriculomegaly observed on MR images and a positive CSF drainage test shifted the consideration to normal-pressure hydrocephalus. Finally, the diagnostic journey concluded when genetic testing and pathological biopsy confirmed ANCL, highlighting the arduous and convoluted nature of this process. Differentiating ANCL from other neurodegenerative disorders, such as Huntington disease and early onset Alzheimer disease, is imperative.

### 4.5. Adult-onset neuronal ceroid lipofuscinosis *treatment*

The primary management of ANCL involves controlling seizures with antiepileptic drugs; physical therapy to preserve muscle function; rehabilitation to support daily functioning and quality of life; nutritional support; and symptomatic treatment for depression, anxiety, and sleep disturbances. Piracetam is effective for both seizures and ataxia.^[13]^ Some cases have shown that levetiracetam can effectively control epilepsy.^[7]^ The blood-brain barrier limits the effectiveness of many treatments for lysosomal storage disorders in NCL.^[26]^ Identifying mutations that cause disease lays the groundwork for developing targeted treatments, such as enzyme replacement therapies, gene therapies for the brain, cell therapies, and drugs that can adjust defective molecular pathways.^[27]^ Enzyme replacement therapy, stem cell therapy, gene therapy vectors, and pharmacological drugs have all been assessed for their safety and effectiveness in preclinical and clinical studies.^[28,29]^ The first sanctioned treatment was an enzyme administered intracerebroventricularly to target NCL2, which delays symptom progression.^[26]^ Combination treatments may prove beneficial in slowing or halting disease progression. However, it is unlikely that many therapies will partially reverse the condition, and complete reversal is currently considered improbable.

## 5. Conclusions

ANCL exhibits a high degree of clinical heterogeneity, characterized primarily by refractory seizures, progressive cognitive decline, and movement disorders, which need to be differentiated from autoimmune encephalitis, normal-pressure hydrocephalus, and Parkinson syndrome. MRI reveals progressive brain atrophy. Enhanced clinical and imaging insights into ANCL, coupled with its early consideration in the differential diagnosis, can result in improved management and the avoidance of overtreatment. Genetic testing and pathological examination are essential for diagnosis. Further research is crucial to deepen our understanding of pathological mechanisms and develop more effective treatments.

## Acknowledgments

We would like to thank the “Double-First Class” Application Characteristic Discipline of Hunan Province (Pharmaceutical Science) for their support.

## Author contributions

**Data curation:** Huasheng Huang.

**Formal analysis:** Huasheng Huang, Yuqi Liao, Yanni Yu, Liming Cao.

**Methodology:** Liming Cao.

**Resources:** Huasheng Huang.

**Visualization:** Yanni Yu, HuiHui Qin, Yi Zhi Wei.

**Writing – original draft:** Huasheng Huang, Yuqi Liao, Yanni Yu, Liming Cao.

**Writing – review & editing:** Liming Cao.
